# Association of the TSHR Gene Intronic Single Nucleotide Polymorphism rs2268458 With Non-autoimmune Hypothyroidism: A Hospital-Based Cross-Sectional Study

**DOI:** 10.7759/cureus.112850

**Published:** 2026-07-17

**Authors:** M. Usha Rani, Sai Shanmukh Vemparala, Swarnalatha Pakalapati, Dhanunjaya Varma Lakkamraju

**Affiliations:** 1 Physiology, GITAM Institute of Medical Sciences and Research, Visakhapatnam, IND; 2 Physiology, Great Eastern Medical School, Visakhapatnam, IND; 3 Multidisciplinary Research Unit, Andhra Medical College, Visakhapatnam, IND; 4 Human Genetics, Andhra University, Visakhapatnam, IND

**Keywords:** non-autoimmune hypothyroidism, pcr-rflp, sanger sequencing, snp rs2268458, thyroid-stimulating hormone receptor (tshr) gene

## Abstract

Background

Nonautoimmune hypothyroidism is underexplored despite its potential metabolic and muscular complications. This study investigated the thyroid-stimulating hormone receptor (TSHR) intronic single nucleotide polymorphism (SNP) rs2268458 to assess its association with non-autoimmune hypothyroidism in an Indian population.

Methods

A hospital-based cross-sectional study was conducted between May 2023 and July 2024 and included 36 patients with non-autoimmune hypothyroidism, 22 healthy controls, and 13 autoimmune hypothyroid patients (positive controls). Thyroid function (T3, T4, and thyroid-stimulating hormone (TSH)) and anti-thyroid peroxidase (anti-TPO) antibody levels were measured using electrochemiluminescence immunoassay. Genotyping of the TSHR rs2268458 polymorphism was performed using PCR-restriction fragment length polymorphism (PCR-RFLP) and confirmed by Sanger sequencing. Genotype and allele frequencies were analyzed in relation to thyroid biochemical parameters.

Results

The patients showed significantly higher TSH and lower T3 levels than controls (p < 0.05), along with elevated T4 levels. Anti-TPO antibody levels were substantially lower than those observed in autoimmune hypothyroid patients. The TC genotype was the most frequent among patients (52.78%), whereas healthy controls demonstrated a higher frequency of the CC genotype. Genotype distribution differed between patients and controls, with predominance of the TC genotype among patients.

Conclusion

The TSHR intronic SNP rs2268458 may be associated with non-autoimmune hypothyroidism, with predominance of the TC genotype among affected individuals. These findings suggest a possible role of TSHR genetic variation in disease susceptibility independent of overt autoimmunity. Further large-scale and functional studies are required to validate these observations.

## Introduction

Thyroid hormones regulate metabolism and influence the secretion of other hormones [1]. Around 20 million people in the United States and approximately 42 million in India are affected by thyroid disorders [2,3]. The prevalence varies according to geographic, environmental, and demographic factors. Congenital hypothyroidism is particularly important because early diagnosis is essential to prevent neurodevelopmental impairment. A Mumbai-based study reported that 79% of children with thyroid disorders had hypothyroidism [4]. In addition, non-autoimmune hypothyroidism has been associated with complications such as rhabdomyolysis and cardiac dysfunction [5].

Despite the high prevalence of hypothyroidism, the genetic basis of non-autoimmune hypothyroidism remains incompletely understood. Mutations in the thyroid-stimulating hormone receptor (TSHR) gene have been implicated in subclinical hypothyroidism and are thought to contribute to autoimmune thyroid manifestations [6]. TSHR intron 1 spans approximately 106 kb (~56% of the gene) and harbors a putative alternative promoter. The presence of multiple TSHR mRNA isoforms suggests a role for intron 1-mediated alternative splicing in TSHR function and Graves' disease susceptibility, with potential contributions from intronic small RNAs regulating gene expression and immune responses [7]. Intronic variants of TSHR, such as rs2268458, have also been implicated in thyroid dysfunction and Graves' disease across various ethnic groups, including Indian populations [8].

The present study investigated the association between the TSHR intronic single nucleotide polymorphism (SNP) rs2268458 and non-autoimmune hypothyroidism in patients from Andhra Pradesh. Genotypic and allelic analyses, correlated with serum thyroid-stimulating hormone (TSH) levels, were performed to evaluate genetic susceptibility and improve understanding of disease pathogenesis, with potential implications for risk prediction and targeted therapy.

The primary objective of this study was to evaluate the association between the TSHR intronic SNP rs2268458 and non-autoimmune hypothyroidism in an Indian population. The secondary objectives were to compare genotype and allele frequencies among non-autoimmune hypothyroid patients, autoimmune hypothyroid patients, and healthy controls, and to assess their relationship with thyroid biochemical parameters.

## Materials and methods

Study design

This analytical cross-sectional study was conducted at the Multidisciplinary Research Unit (MRU), Andhra Medical College and King George Hospital, Visakhapatnam, India, between May 2023 and July 2024. The study aimed to evaluate the association between non-autoimmune hypothyroidism and the TSHR gene polymorphism rs2268458. The TSHR gene (Gene ID: 7253) is located on chromosome 14 (NC_000014.9: 80,955,621-81,146,306), and the investigated SNP was rs2268458.

Study participants and biochemical parameters

A total of 73 individuals presenting with symptoms suggestive of hypothyroidism were recruited consecutively from the departments of endocrinology and internal medicine. Participants aged 18-70 years were eligible for inclusion. Of the 73 participants initially recruited, two were excluded because of incomplete laboratory data, leaving 71 participants for the final analysis.

Participants were classified into three groups based on clinical assessment and biochemical evaluation, including serum TSH, triiodothyronine (T3), thyroxine (T4), and anti-thyroid peroxidase (anti-TPO) antibody levels. The non-autoimmune hypothyroidism group (n = 36) comprised participants with elevated serum TSH (>4.50 mIU/L), reduced or normal T3 and T4 levels, and negative anti-TPO antibodies (<30 IU/mL). The autoimmune hypothyroidism group (positive controls; n = 13) included participants with hypothyroidism and positive anti-TPO antibody levels (≥30 IU/mL). The healthy control group (n = 22) consisted of individuals without clinical features of thyroid disease and with normal thyroid function tests and negative anti-TPO antibody levels [9-11].

Serum TSH, T3, T4, and anti-TPO antibody levels were measured using electrochemiluminescence immunoassay (ECLIA) on a Roche Cobas e411 analyzer (Roche Diagnostics, Mannheim, Germany). The laboratory reference ranges were as follows: TSH: 0.40-4.50 mIU/L; T3: 0.92-2.79 nmol/L; T4: 4.87-11.72 μg/dL; and anti-TPO antibody: <30 IU/mL. Some hypothyroid participants were receiving levothyroxine therapy at the time of recruitment; however, treatment duration and dosage were not systematically recorded.

Samples with incomplete genotyping or poor-quality sequencing results were excluded from subgroup genetic analyses where applicable.

DNA isolation and SNP analysis

Peripheral blood samples were collected in K₂EDTA (dipotassium ethylenediaminetetraacetic acid) vials for DNA extraction. Genomic DNA was extracted using the QIAamp DNA Mini Kit (Qiagen, Hilden, Germany). DNA concentration and purity were assessed by spectrophotometry. The target region of the TSHR gene was amplified by polymerase chain reaction (PCR) using a forward primer (5′-CCAGCAGAGGGAGCACAA-3′) and a reverse primer (5′-AGTCTCTTGCTGCTCTATTCTCTA-3′). The total reaction volume was 50 μL, containing 25 μL EmeraldAmp GT PCR Master Mix, 1 μL of each primer, 200 ng genomic DNA, and diethylpyrocarbonate (DEPC)-treated water. PCR amplification consisted of an initial denaturation at 98°C for five minutes, followed by 35 cycles of denaturation at 98°C for 10 seconds, annealing at 57°C for 30 seconds, and extension at 72°C for 30 seconds, with a final extension at 72°C for five minutes. PCR products were confirmed by electrophoresis on a 1.5% agarose gel, yielding the expected 415-bp amplicon.

Restriction fragment length polymorphism (RFLP) and Sanger sequencing

The 415-bp PCR amplicon was digested overnight with 10 U of AluI restriction enzyme (Thermo Fisher Scientific, Waltham, MA) at 37°C [12]. Digested products were separated on a 3.5% low-electroendosmosis (EEO) agarose gel using a 50-bp DNA ladder and visualized under ultraviolet illumination. The expected restriction fragment patterns were TT genotype (333 bp and 82 bp), CC genotype (357 bp, 275 bp, 82 bp, and 58 bp), and CT genotype (357 bp, 333 bp, 275 bp, 82 bp, and 58 bp). PCR amplicons were subsequently subjected to Sanger sequencing using a 3730XL DNA Analyzer to validate PCR-RFLP findings [13-15]. Chromatogram (.abi) files were analyzed using Geneious Prime [16,17] and Chromas software [18,19].

Statistical analysis

Statistical analysis was performed using IBM SPSS Statistics version 26.0 (IBM Corp., Armonk, NY) [20-22]. Continuous variables are presented as mean ± standard deviation (SD). Comparisons among continuous variables were performed using one-way analysis of variance (ANOVA), followed by appropriate pairwise comparisons. Categorical variables, including genotype and allele frequencies, were analyzed using Fisher's exact test because of the small expected cell counts. Odds ratios (ORs) with 95% confidence intervals (CIs) were calculated to estimate the strength of association. Binary logistic regression analysis was additionally performed to evaluate the association between TSHR genotypes and non-autoimmune hypothyroidism after adjustment for age and sex. A p-value <0.05 was considered statistically significant.

Ethics and approvals

The study was approved by the Institutional Ethics Committee, Andhra Medical College, Visakhapatnam (Approval No. 123/IEC AMC/MAY 2023). Written informed consent was obtained from all participants in their local language before enrollment in accordance with the principles of the Declaration of Helsinki. All biological samples were anonymized before laboratory analysis to ensure participant confidentiality.

## Results

Clinical characteristics and demographics

A total of 71 participants were included in the final analysis, comprising 36 non-autoimmune hypothyroid subjects (test group), 22 healthy controls, and 13 autoimmune hypothyroid subjects (positive controls). The mean age of the test group (42.94 ± 12.82 years) was significantly higher than that of healthy controls (29.00 ± 6.29 years; p < 0.0001), but did not differ significantly from the positive control group (38.07 ± 12.60 years; p = 0.691).

A marked female predominance was observed in the test group (male:female = 4:32). The sex distribution approached statistical significance when compared with healthy controls (p = 0.051), but did not differ significantly from the positive control group (p = 0.179).

Comorbid conditions such as diabetes mellitus and hypertension were reported in a small proportion of participants in the test and positive control groups but were absent in healthy controls. However, these differences were not statistically significant between groups. The demographic and clinical characteristics of the study participants are summarized in Table 1.

**Table 1:** Clinical characteristics of the study participants. Comparison of demographic and clinical characteristics among the study groups. Continuous variables were analyzed using one-way ANOVA, and categorical variables were analyzed using Fisher’s exact test. Statistical significance was considered at p < 0.05. DM: diabetes mellitus; HTN: hypertension.

Demography	Test (n = 36)	Controls (n = 22)	t/χ2	P-value	Positive controls (n = 13)	t/χ2	P-value
Age	42.94 ± 12.82	29.00 ± 6.29	4.754	<0.0001	38.07 ± 12.60	1.245	0.691
Sex (M/F)	4/32	7/15	3.810	0.051	0/13	1.809	0.179
DM (Yes/No)	5/31	0/22	3.344	0.067	2/13	0.003	0.958
HTN (Yes/No)	5/31	0/22	3.344	0.067	2/13	0.003	0.958

Biochemical parameters analysis

Biochemical analysis showed that serum triiodothyronine (T3) levels were significantly lower in the test group (1.07 ± 0.34 nmol/L) than in healthy controls (1.54 ± 0.45 nmol/L; p < 0.001). Serum thyroxine (T4) levels were significantly higher in the test group (10.79 ± 3.47 µg/dL) than in healthy controls (6.77 ± 1.67 µg/dL; p < 0.001). Serum TSH levels were also significantly higher in the test group (4.19 ± 5.79 mIU/L) than in healthy controls (1.85 ± 0.57 mIU/L; p = 0.021).

Anti-TPO antibody concentrations were markedly elevated in autoimmune hypothyroid patients (positive controls) (570.62 ± 320.28 IU/mL; p < 0.001), confirming their autoimmune status. In contrast, non-autoimmune hypothyroid patients demonstrated substantially lower anti-TPO antibody levels, although these remained slightly higher than those observed in healthy controls. As some hypothyroid participants were receiving levothyroxine therapy at the time of recruitment, thyroid hormone concentrations should be interpreted with caution. However, treatment duration and dosage were not systematically recorded. The biochemical findings are summarized in Table 2.

**Table 2:** Comparative analysis of biochemical parameters among the study groups. Comparison of thyroid biochemical parameters among non-autoimmune hypothyroid patients (test group), healthy controls, and autoimmune hypothyroid patients (positive controls). Continuous variables were analyzed using one-way ANOVA, followed by pairwise comparisons where appropriate. Statistical significance was considered at p < 0.05. T3: triiodothyronine; T4: thyroxine; TSH: thyroid-stimulating hormone; Anti-TPO: anti-thyroid peroxidase; Ab: antibody.

Observations	Test (n = 36)	Controls (n = 22)	t-value	p-value	Positive controls (n = 13)	t-value	p-value
T3	1.07 ± 0.34	1.54 ± 0.45	-4.21	<0.001	1.17 ± 0.88	-0.40	0.696
T4	10.79 ± 3.47	6.77 ± 1.67	5.92	<0.001	10.78 ± 3.83	0.01	0.993
TSH	4.19 ± 5.79	1.85 ± 0.57	2.40	0.021	3.53 ± 3.21	0.50	0.618
Anti-TPO Ab	2.98 ± 4.90	1.08 ± 1.00	2.25	0.030	570.62 ± 320.28	-6.39	<0.001

DNA amplification and genotyping

After amplification, PCR products were resolved on a 2% agarose gel, and a clear 415-bp band representing the target fragment was detected in all successfully amplified samples.

RFLP results analysis

After overnight digestion with AluI, the restriction fragments were resolved on a 3.5% low-EEO agarose gel using a 50-bp DNA ladder. RFLP genotypes were determined based on the characteristic banding patterns observed under UV illumination.

**Figure 1:**
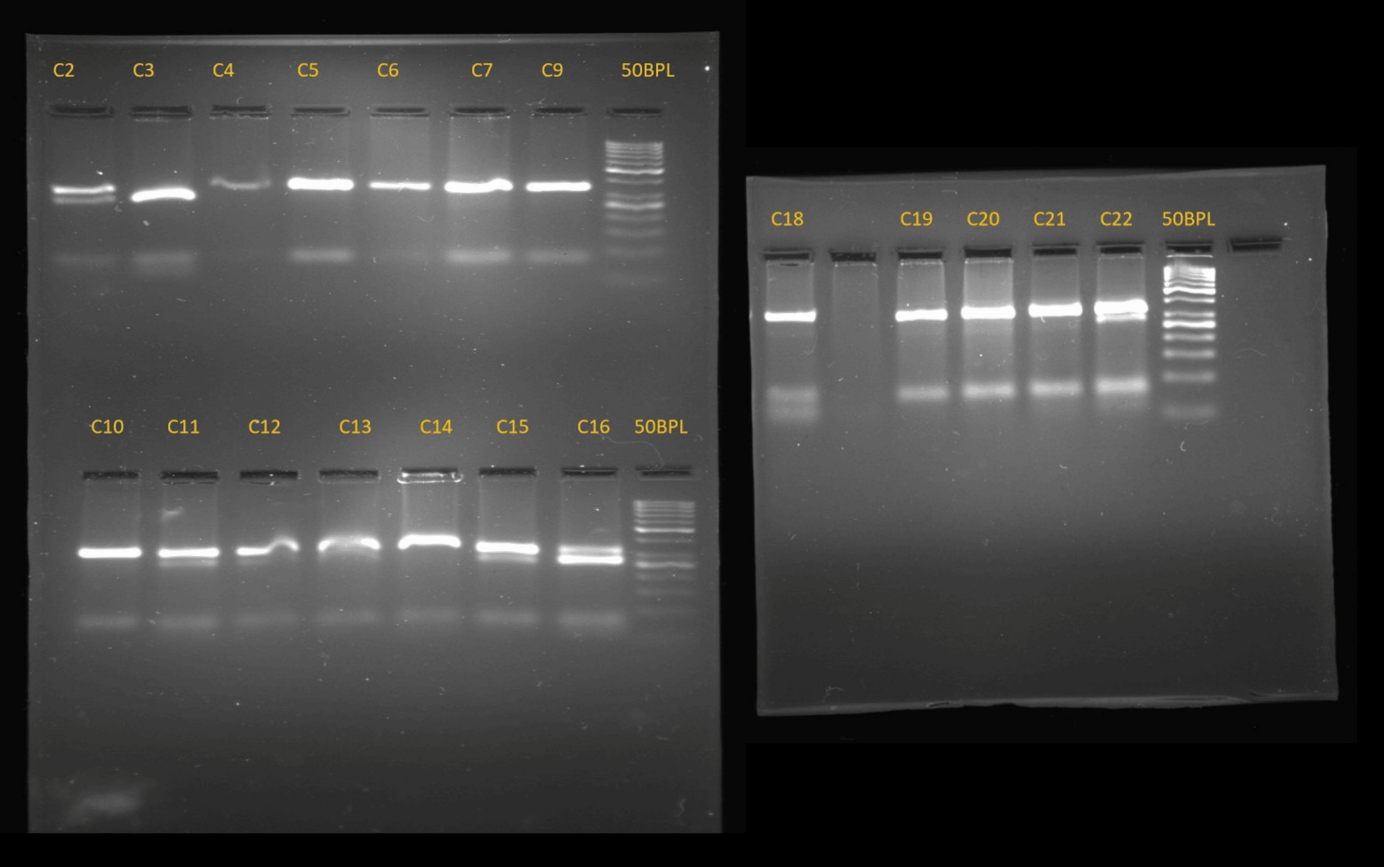
PCR-RFLP analysis of healthy control samples. Representative PCR-RFLP banding patterns of healthy control samples. CC genotypes were identified in samples C3, C16, and C18; TT genotypes in samples C4, C5, C6, C7, C9, C10, C14, C19, C20, and C21; and CT genotypes in samples C2, C11, C12, C13, C15, and C22. Expected fragment patterns were TT (333 and 82 bp), CC (357, 275, 82, and 58 bp), and CT (357, 333, 275, 82, and 58 bp). PCR: polymerase chain reaction; RFLP: restriction fragment length polymorphism.

**Figure 2:**
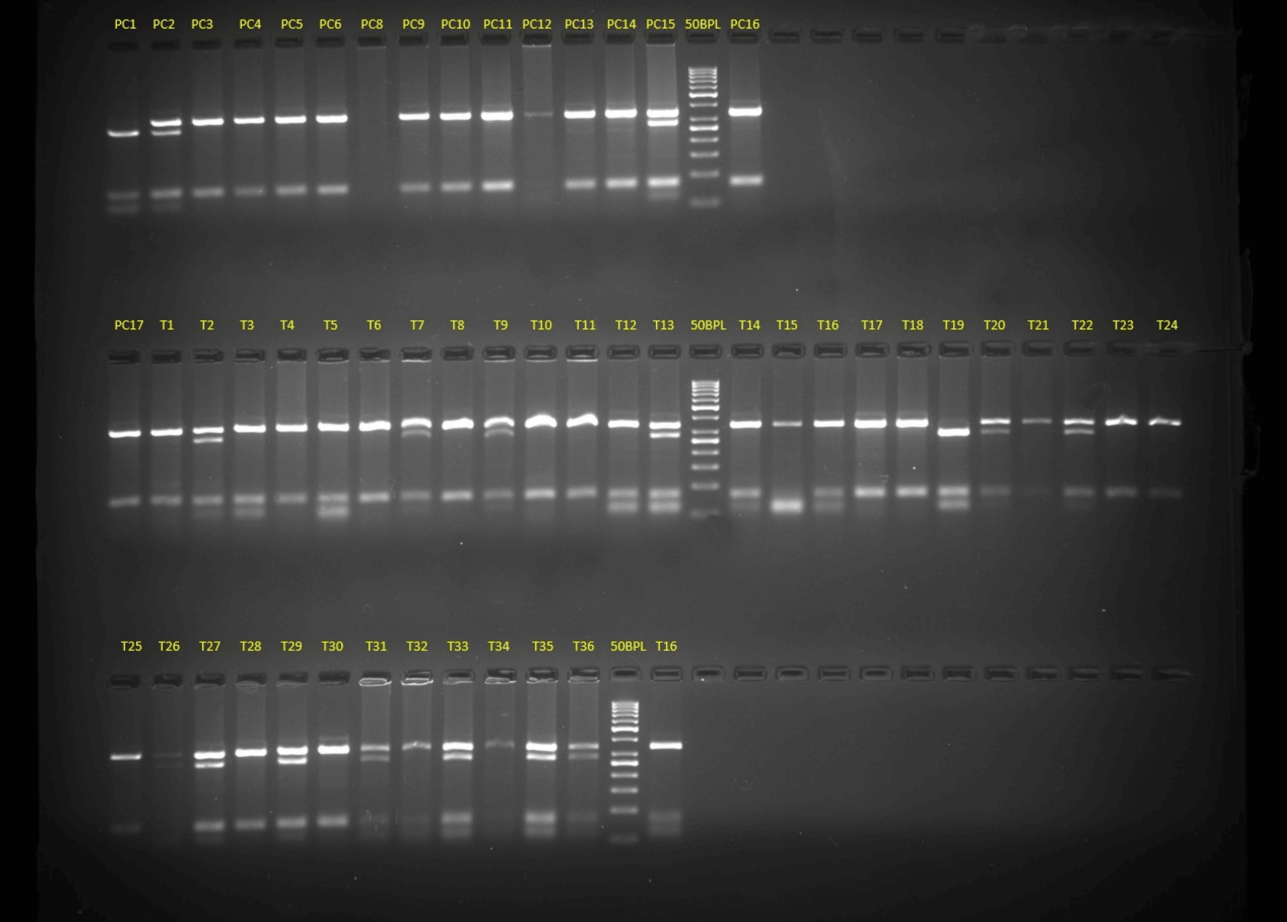
PCR-RFLP analysis of test and positive control samples. Representative PCR-RFLP banding patterns of non-autoimmune hypothyroid (test) and autoimmune hypothyroid (positive control) samples. Among the positive controls, PC2 demonstrated the CT genotype, whereas PC1, PC9, and PC15 demonstrated the CC genotype. The remaining positive control samples demonstrated the TT genotype. Among the test samples, CC genotypes were observed in T15 and T19; TT genotypes in T1, T4, T6, T8, T10, T11, T17, T18, T21, T23, T24, T25, T26, T28, and T30; and CT genotypes in T2, T3, T5, T7, T9, T12, T13, T14, T16, T20, T22, T27, T29, T31, T32, T33, T34, T35, and T36. Expected fragment patterns were TT (333 and 82 bp), CC (357, 275, 82, and 58 bp), and CT (357, 333, 275, 82, and 58 bp). PCR: polymerase chain reaction; RFLP: restriction fragment length polymorphism.

PCR-RFLP analysis revealed three distinct banding patterns corresponding to the TT, CC, and CT genotypes (Figure 1). The homozygous TT genotype produced two fragments of 333 bp and 82 bp and was observed in 15 samples (T1, T4, T6, T8, T10, T11, T17, T18, T21, T23, T24, T25, T26, T28, and T30). The homozygous CC genotype produced four fragments of 357 bp, 275 bp, 82 bp, and 58 bp and was observed in two samples (T15 and T19). The heterozygous CT genotype demonstrated all five expected fragments (357 bp, 333 bp, 275 bp, 82 bp, and 58 bp) and was identified in 19 samples (T2, T3, T5, T7, T9, T12, T13, T14, T16, T20, T22, T27, T29, T31, T32, T33, T34, T35, and T36). Representative RFLP patterns for healthy controls, non-autoimmune hypothyroid patients, and autoimmune hypothyroid patients are shown in Figures 1, 2.

Sanger sequencing analysis

**Figure 3:**
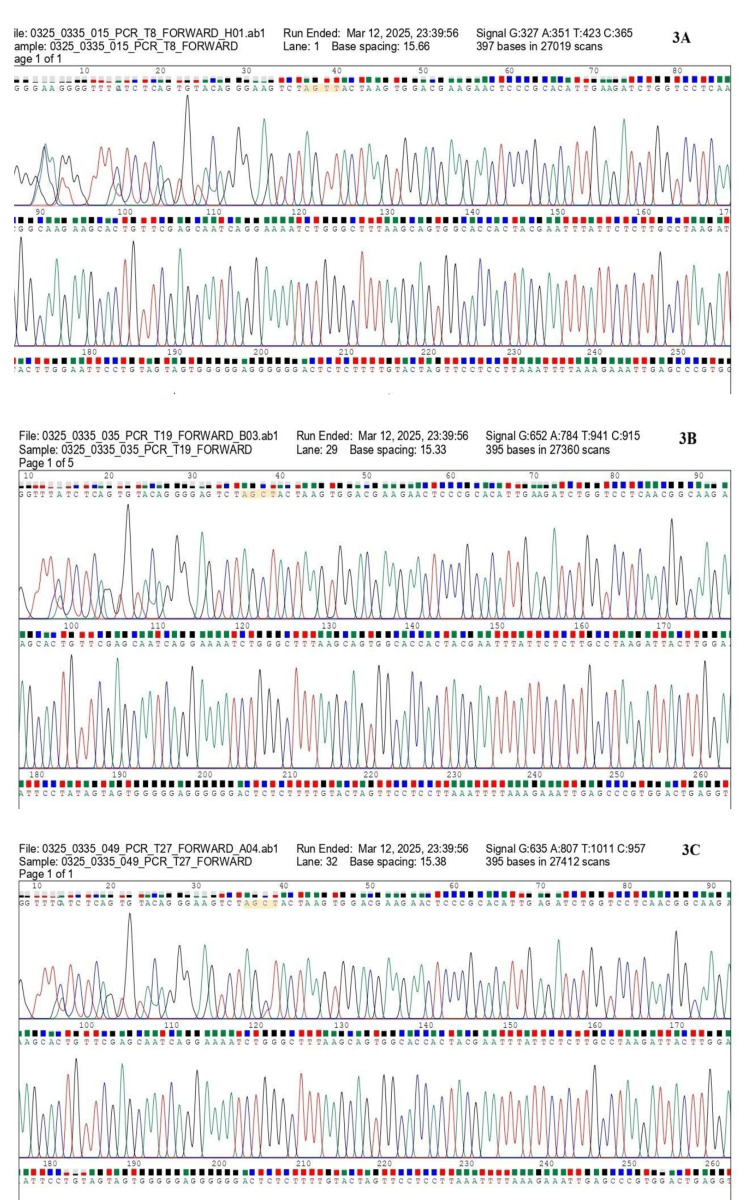
Representative Sanger sequencing chromatograms of test samples. Representative Sanger sequencing chromatograms of non-autoimmune hypothyroid (test) samples. Figure 3A shows the TT genotype (sample T8), Figure 3B shows the CC genotype (sample T19), and Figure 3C shows the CT genotype (sample T27). The rs2268458 polymorphic site is highlighted.

**Figure 4:**
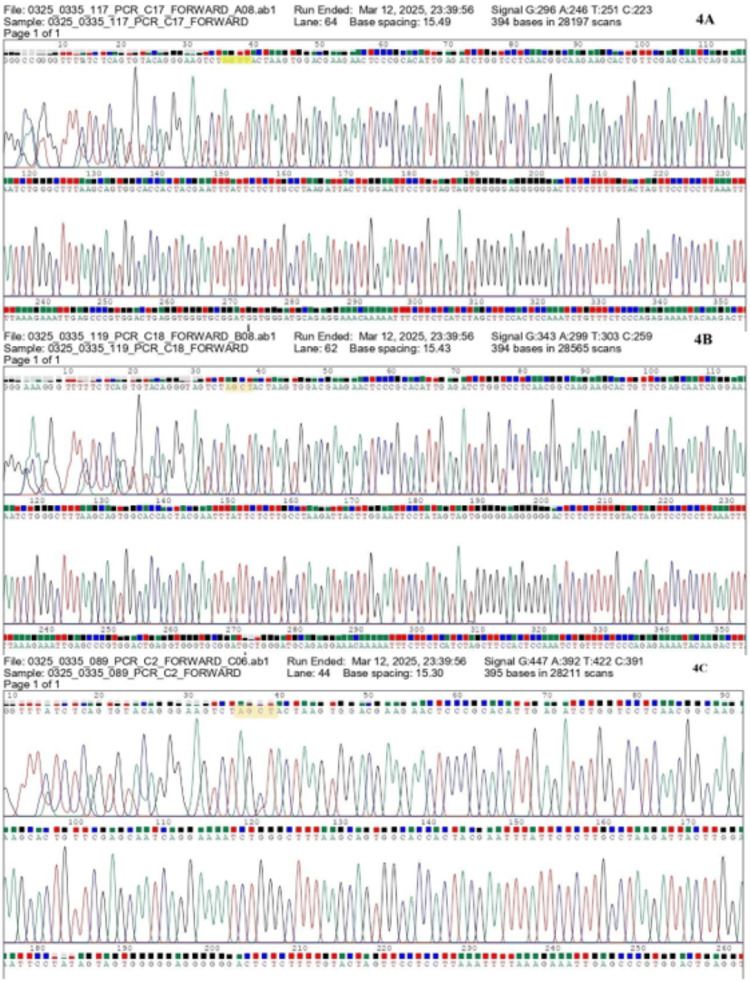
Representative Sanger sequencing chromatograms of healthy control samples. Representative Sanger sequencing chromatograms of healthy control samples. Figure 4A shows the TT genotype (sample C17), Figure 4B shows the CC genotype (sample C2), and Figure 4C shows the CT genotype (sample C18). The rs2268458 polymorphic site is highlighted.

**Figure 5:**
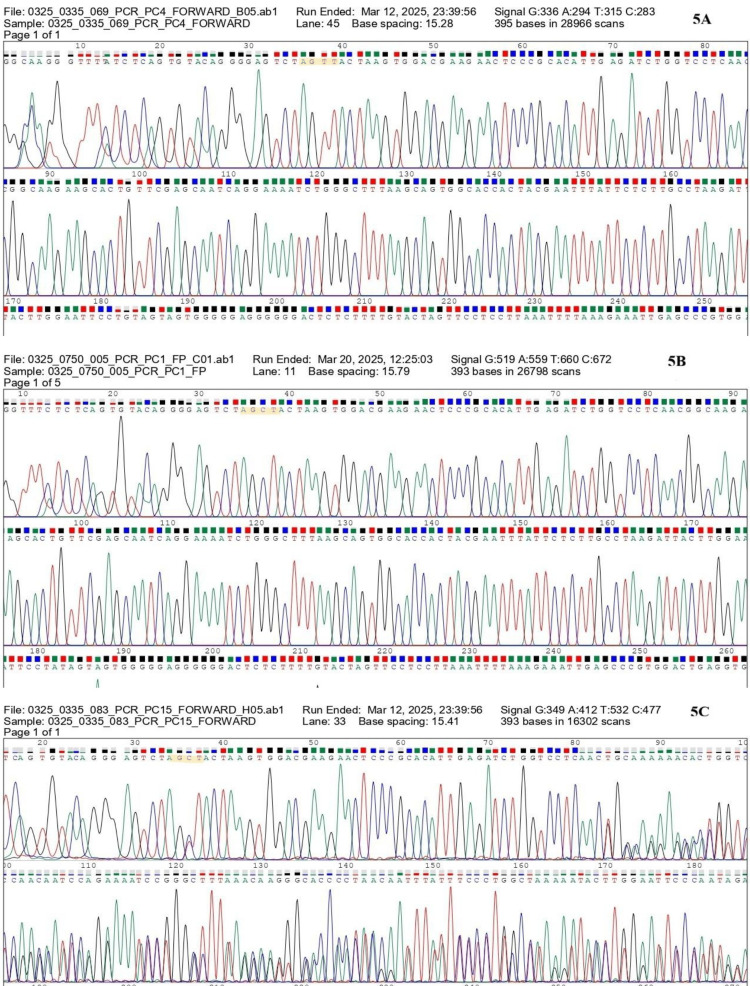
Representative Sanger sequencing chromatograms of positive control samples. Representative Sanger sequencing chromatograms of autoimmune hypothyroid (positive control) samples. Figure 5A shows the TT genotype (sample PC1), Figure 5B shows the CC genotype (sample PC4), and Figure 5C shows the CT genotype (sample PC15). The rs2268458 polymorphic site is highlighted.

Sanger sequencing was performed to validate the PCR-RFLP genotyping results by confirming the nucleotide variation at the rs2268458 locus. Sequence chromatograms and multiple sequence alignments were analyzed using Geneious Prime and Chromas software. Only high-quality chromatograms were included in the final sequence analysis (Figures 3-5).

**Figure 6:**
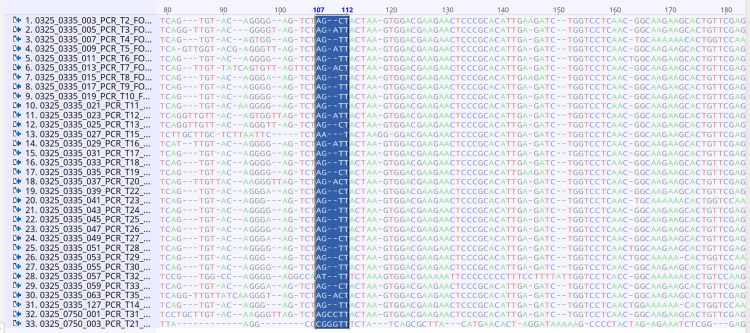
Multiple sequence alignment of test samples. Multiple sequence alignment of representative test samples. An adenine (A) insertion was observed at the highlighted polymorphic site in seven of the 33 high-quality sequences included in the alignment. The biological significance of this observation requires further investigation.

**Figure 7:**
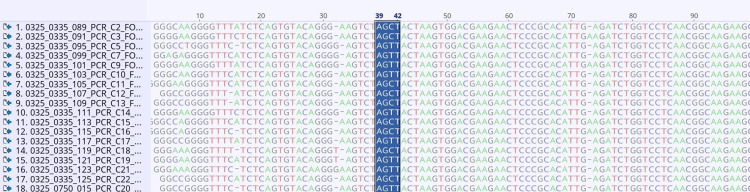
Multiple sequence alignment of healthy control samples. Multiple sequence alignment of representative healthy control samples. The highlighted region indicates the TSHR rs2268458 polymorphic site.

**Figure 8:**
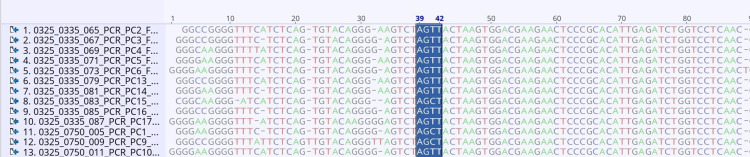
Multiple sequence alignment of positive control samples. Multiple sequence alignment of representative autoimmune hypothyroid (positive control) samples. The highlighted region indicates the TSHR rs2268458 polymorphic site.

Multiple sequence alignments of test samples, healthy controls, and positive controls are presented in Figures 6-8, respectively. Sequence alignments were generated from high-quality Sanger sequencing chromatograms to visualize nucleotide variation at the TSHR rs2268458 locus.

Genotypic and allelic analysis

Comparison Between Test Group and Healthy Controls

One healthy control sample was excluded from genotype analysis because of incomplete genotyping results. Genotypic analysis of the TSHR rs2268458 polymorphism revealed that the heterozygous CT genotype was the most frequent in the test group (52.78%), followed by the TT genotype (41.67%) and CC genotype (5.56%). In contrast, healthy controls demonstrated a higher frequency of the CC genotype (33.33%) and a lower frequency of the CT genotype (19.05%), whereas the frequency of the TT genotype was comparable between groups. The frequency of the T allele was higher in the test group (68.06%) than in healthy controls (57.14%); however, this difference was not statistically significant (p = 0.243). A summary of these findings is presented in Table 3.

**Table 3:** Genotype and allele distribution of the TSHR rs2268458 polymorphism between the test group and healthy controls. Distribution of TSHR rs2268458 genotype and allele frequencies among non-autoimmune hypothyroid patients (test group) and healthy controls. Genotype and allele frequencies were compared using Fisher's exact test. One healthy control sample was excluded because of incomplete genotyping results. Odds ratios (ORs) and 95% confidence intervals (CIs) were calculated using the TT genotype as the reference (REF). Statistical significance was defined as p < 0.05.

Model		Test (N = 36)	%	Controls (n = 21)	%	χ2	OR (95% CI)	P-value
Genotypes	CC	2	5.56%	7	33.33%	10.3284 (0.0013)	0.1905 (0.0327 to 1.1110)	0.0653
	CT	19	52.78%	4	19.05%		3.1667 (0.8269 to 12.1264)	0.0925
	TT	15	41.67%	10	47.62%		REF	
Alleles	C	23	31.94%	18	42.86%	1.3716 (0.2415)	0.6259 (0.2850 to 1.3745)	0.2430
	T	49	68.06%	24	57.14%			

To account for the significant age difference between study groups, binary logistic regression analysis was performed after adjustment for age and sex. The CT genotype showed higher odds of non-autoimmune hypothyroidism than the TT genotype (adjusted OR = 3.14, 95% CI: 0.61-16.24); however, this association did not reach statistical significance (p = 0.172). Increasing age was independently associated with non-autoimmune hypothyroidism (adjusted OR = 1.16 per year, 95% CI: 1.05-1.29; p = 0.002), whereas sex was not a significant predictor (adjusted OR = 0.88, 95% CI: 0.09-8.79; p = 0.913). The results of the adjusted logistic regression analysis are presented in Table 4. The genotype distribution among healthy controls deviated from Hardy-Weinberg equilibrium (χ² = 7.85, p = 0.005).

**Table 4:** Binary logistic regression analysis of factors associated with non-autoimmune hypothyroidism. Binary logistic regression was performed to evaluate the association between the TSHR rs2268458 genotype and non-autoimmune hypothyroidism after adjustment for age and sex. The TT genotype was used as the reference category for genotype comparisons. Statistical significance was considered at p < 0.05; statistically significant values are indicated by an asterisk (*). OR: odds ratio; CI: confidence interval.

Variable	Adjusted OR	95% CI	p-value
CT genotype (vs. TT)	3.14	0.61-16.24	0.172
Age (per year increase)	1.16	1.05-1.29	0.002*
Female sex (vs. male)	0.88	0.09-8.79	0.913

Comparison Between Test and Positive Controls

When the test group was compared with autoimmune hypothyroid patients (positive controls), the heterozygous CT genotype was more frequent in the test group (52.78%) than in the positive control group (15.38%), corresponding to significantly higher odds of non-autoimmune hypothyroidism (OR = 6.33, 95% CI: 1.20-33.39; p = 0.0295). In contrast, the TT genotype was more common in the positive control group (76.92%) than in the test group (41.67%). Although the T allele predominated in both groups, the difference in allele frequencies was not statistically significant (OR = 2.58, 95% CI: 0.80-8.36; p = 0.1136). The genotype and allele distributions are summarized in Table 5.

**Table 5:** Genotype allele distribution between test and positive control groups. Distribution of TSHR rs2268458 genotype and allele frequencies among non-autoimmune hypothyroid patients (test group) and autoimmune hypothyroid patients (positive controls). Genotype and allele frequencies were compared using Fisher's exact test. Odds ratios (ORs) with 95% confidence intervals (CIs) were calculated using the TT genotype as the reference (REF). Statistical significance was defined as p < 0.05.

Model		Test (n = 36)	%	Positive controls (n = 13)	%	χ2	OR (95% CI)	P-value
Genotypes	CC	2	5.56%	1	7.69%	5.5142 (0.0189)	1.3333 (0.1062 to 16.7439)	0.8237
	CT	19	52.78%	2	15.38%		6.3333 (1.2014 to 33.3858)	0.0295
	TT	15	41.67%	10	76.92%		REF	
Alleles	C	23	31.94%	4	15.38%	2.6243 (0.1052)	2.5816 (0.7973 to 8.3591)	0.1136
	T	49	68.06%	22	84.62%			

## Discussion

Among the study participants, non-autoimmune hypothyroid (NAH) patients were older and predominantly female compared with healthy controls. Diabetes mellitus and hypertension were present in a small proportion of NAH patients but were absent in controls. Biochemical analysis revealed that NAH patients exhibited altered thyroid hormone profiles compared with controls. Anti-TPO antibody concentrations were significantly lower in the test group than in autoimmune hypothyroid patients, supporting their classification as non-autoimmune hypothyroidism. Notably, serum T4 levels were elevated in NAH patients. This finding may partly reflect levothyroxine therapy, as some hypothyroid participants were receiving treatment at the time of recruitment, although treatment duration and dosage were not systematically recorded. Altered peripheral thyroid hormone metabolism may also have contributed to this observation.

The TSHR gene encodes the G protein-coupled receptor for TSH and plays an essential role in thyroid follicular cell activation, growth, and hormone synthesis. Pathogenic variants of TSHR have been well documented in Graves’ disease and Hashimoto’s thyroiditis, which represent major autoimmune thyroid disorders [23,24]. The intronic SNP rs2268458, located within intron 1 of the TSHR gene, has been implicated in transcriptional regulation and alternative splicing. Stefan et al. [23] first reported an association between this SNP and Graves’ disease, and subsequent studies linked it to altered TSHR expression patterns [24,25]. However, relatively little attention has been given to its possible role in non-autoimmune hypothyroidism, particularly in the context of intronic regulatory variants. Although intronic SNPs do not alter protein-coding sequences, they may exert regulatory effects through modulation of splice sites, enhancer or silencer elements, and transcription factor binding motifs. Our findings suggest that the rs2268458 polymorphism may contribute to genetic susceptibility to non-autoimmune hypothyroidism; however, this observation requires confirmation in larger studies.

Genotypic analysis revealed a predominance of the heterozygous TC genotype among NAH patients, whereas healthy controls showed a higher frequency of the CC genotype. The TT genotype frequency was comparable between NAH patients and controls, but was more prevalent in autoimmune hypothyroid cases. Although a higher prevalence of the T allele was observed in NAH patients, the difference was not statistically significant compared with controls.

To account for the significant age difference between study groups, a binary logistic regression analysis adjusted for age and sex was performed. Although the adjusted odds ratio for the CT genotype increased compared with the unadjusted analysis, the association remained statistically non-significant. In contrast, increasing age was independently associated with non-autoimmune hypothyroidism, indicating that age acted as an important confounding factor in the observed genotype distribution.

These observations are consistent with earlier studies that have highlighted the functional relevance of non-coding genetic variants in endocrine disorders. Yamaguchi et al. [26] demonstrated that intronic variants in G protein-coupled receptor genes can influence splicing efficiency and cell-surface receptor density, thereby affecting hormone responsiveness. Similarly, Dechairo et al. [24] have associated TSHR variants with susceptibility to autoimmune thyroid diseases, although most previous studies focused on exonic or promoter-region polymorphisms rather than deep intronic variants.

The distinctive contribution of the present study lies in its focus on non-autoimmune hypothyroidism, a condition that remains relatively underexplored in genetic studies because of the predominant emphasis on autoimmune thyroid disorders. The observed differences in genotype distribution among NAH patients, together with altered biochemical profiles such as elevated T4 and reduced T3 levels, suggest that NAH may involve distinct pathophysiological mechanisms rather than simply representing an early stage of autoimmune hypothyroidism. These findings indicate the potential involvement of intronic regulatory variants in NAH susceptibility.

Clinical implications

The observed association of rs2268458 with NAH may have potential clinical implications. Although the present findings suggest that the rs2268458 polymorphism may contribute to susceptibility to non-autoimmune hypothyroidism, its clinical utility as a genetic marker remains uncertain. Validation in larger multicenter cohorts and functional studies is required before consideration of routine clinical application.

Limitations

The present study has several limitations. First, the relatively small sample size and single-center design may limit the generalizability of the findings. Second, no formal a priori sample size calculation was performed. Third, some hypothyroid participants were receiving levothyroxine therapy at the time of recruitment; however, treatment duration and dosage were not systematically recorded, which may have influenced thyroid hormone measurements. Fourth, the genotype distribution among healthy controls deviated from Hardy-Weinberg equilibrium. This finding should be interpreted cautiously given the limited number of healthy controls and requires confirmation in larger independent cohorts. Finally, although binary logistic regression was performed to adjust for age and sex, residual confounding cannot be excluded. Future multicenter studies involving larger and ethnically diverse populations, together with functional investigations of the rs2268458 polymorphism, are warranted to validate these findings and clarify their biological significance.

## Conclusions

This study suggests a possible association between the TSHR intronic SNP rs2268458 and non-autoimmune hypothyroidism in an Indian cohort, with a predominance of the CT genotype among affected individuals. These findings indicate that genetic variation within the TSHR gene may contribute to susceptibility to non-autoimmune hypothyroidism independent of overt autoimmunity. However, the association remained non-significant after adjustment for age and sex, highlighting the influence of demographic factors and the need for cautious interpretation. The relatively small sample size, single-center design, and cross-sectional nature of the study further limit the generalizability of the findings. Larger multicenter studies and functional investigations are required to validate these observations and to clarify the biological role of the rs2268458 polymorphism in non-autoimmune hypothyroidism.
